# Fabrication of Glass Diaphragm Based Fiber-Optic Microphone for Sensitive Detection of Airborne and Waterborne Sounds

**DOI:** 10.3390/s22062218

**Published:** 2022-03-13

**Authors:** Gaomi Wu, Xinyu Hu, Xin Liu, Zhifei Dong, Yan Yue, Chen Cai, Zhi-mei Qi

**Affiliations:** 1 Aerospace Information Research Institute, Chinese Academy of Sciences, Beijing 100190, China; gaomi_wu@126.com (G.W.); huxinyu19@mails.ucas.ac.cn (X.H.); liuxin182@mails.ucas.ac.cn (X.L.); dongzhifei18@mails.ucas.ac.cn (Z.D.); yueyan20@mails.ucas.as.cn (Y.Y.); caichen@aircas.ac.cn (C.C.); 2 University of Chinese Academy of Sciences, Beijing 100049, China

**Keywords:** fiber-optic microphone, wheel-like glass diaphragm, high sensitivity, good thermal stability, underwater sound detection

## Abstract

A glass-diaphragm microphone was developed based on fiber-optic Fabry-Perot (FP) interferometry. The glass diaphragm was shaped into a wheel-like structure on a 150-μm-thick glass sheet by laser cutting, which consists of a glass disc connected to an outer glass ring by four identical glass beams. Such a structural diaphragm offers the microphone an open air chamber that reduces air damping and increases sensitivity and results in a cardioid direction pattern for the microphone response. The prepared microphone operates at 1550 nm wavelength, showing high stability in a range of temperature from 10 to 40 °C. The microphone has a resonance peak at 1152 Hz with a quality factor of 21, and its 3-dB cut-off frequency is 32 Hz. At normal incidence of 500 Hz sound, the pressure sensitivity of the microphone is 755 mV/Pa and the corresponding minimum detectable pressure is 251 μPa/Hz^1/2^. In addition to the above characteristics of the microphone in air, a preliminary investigation reveals that the microphone can also work stably under water for a long time due to the combination of the open-chamber and fiber-optic structures, and it has a large signal-to-noise ratio in response to waterborne sounds. The microphone prepared in this work is simple, inexpensive, and electromagnetically robust, showing great potential for low-frequency acoustic detection in air and under water.

## 1. Introduction

Acoustic detectors such as microphones and hydrophones are important information-accessing tools with widespread applications in a large variety of fields, including voice communication, environmental noise monitoring, detection and tracking of aerial and underwater vehicles [1], bearing fault diagnosis, wind turbine measurements [2], oil and gas pipeline leakage monitoring [3], and photoacoustic spectroscopy [4,5,6,7,8,9]. The most common acoustic detectors are electret condenser microphones, which are now easily available on the market. However, the traditional electret microphones have low sensitivity and weak anti-electromagnetic interference ability, and their metal diaphragms are easily corroded slowly in air. These shortcomings of electret microphones have caused certain limitation to their applications. As a potential alternative to electret condenser microphones, optical microphones (especially fiber-optic microphones) have currently attracted more and more attention due to their high sensitivity and good compatibility with metal-free diaphragms as well as intrinsic immunity to electromagnetic interference (EMI) [10,11,12]. The EMI immunity offers optical microphones high reliability in harsh electromagnetic environment and allows them to work not only in air but also under water [13].

According to the recent literatures, the optical microphones are generally fabricated with different diaphragms including silver [14], aluminum [15], glass [16], silicon [17,18,19], silicon nitride [20], polymer [21,22], and graphene [23,24]. These diaphragms are often circular, and their edges are clamped on the endface of a cylindrical air chamber to make the chamber closed. The closed air chamber would provide air damping to the diaphragm, leading to attenuation of the vibration amplitude of the diaphragm. Of the above materials, glass diaphragm presents series of advantages such as low cost, uniform thickness, small coefficient of thermal expansion, strong resistance to chemical corrosion, and good vibration behavior without prestressing. Moreover, glass diaphragms with the specially designed geometries can be easily prepared by laser cutting using commercial thin-film glass plates. For these reasons, glass diaphragm presents some unique advantages over the above-mentioned diaphragms.

In this work, we developed a multifunctional optical microphone by combining a fiber-optic Fabry–Perot interferometer (FPI) transducer with a glass diaphragm. The glass diaphragm was designed as the wheel-shaped geometry, consisting of the central glass disc connected with the outer glass ring through four identical glass beams. To protect the diaphragm from damage, the outer ring was chemically bonded to a thick glass ring of the same diameter. The fiber-optic microphone is prepared by assembling both the wheel-shaped glass diaphragm and a single-mode quartz fiber into the custom holder and the distance from the fiber tip to the diaphragm center is controlled. The resulting microphone contains an open air chamber that reduces air damping and increases sensitivity and also enables the device to work under water as a hydrophone. Compared with existing fiber-optic microphones with smaller wheel-shaped diaphragms to operate at resonant frequencies in the high frequency range [25,26], the glass diaphragm designed in this work is larger, allowing the microphone to operate in the flat low frequency range, not just around the resonant peak. Moreover, the microphone response shows high directionality. In the following sections, simulation and design and preparation of the wheel-shaped glass diaphragm are described, and the microphone fabrication process is clearly shown, and the performance characterization of the prepared microphone is emphasized. To the best of our knowledge, this is the first glass diaphragm-based acoustic detector with dual functions of microphone and hydrophone.

## 2. Materials and Methods

### 2.1. Simulation of the Wheel-Shaped Glass Diaphragm

**Figure 1:**
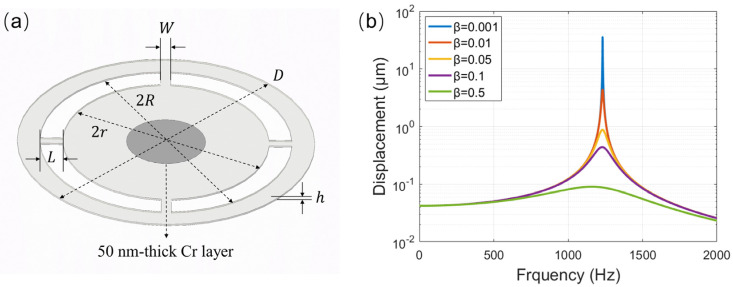
(**a**) Schematic diagram of the wheel-shaped glass diaphragm; (**b**) the calculated frequency responses of the wheel-shaped glass diaphragm with *W* = 2.0 and *L* = 2.3 mm at different damping coefficients.

Figure 1a schematically shows the wheel-shaped glass diaphragm designed in this work. Such structural diaphragm can provide an open air chamber with four front ports for the microphone. These ports allow the sound wave to enter the chamber to interact with the backside of the diaphragm, resulting in a force difference between the front and back surface of the diaphragm, and this force difference can significantly affects the response directionality of the microphone. According to the fundamental theory of acoustics [27], the vibration of the wheel-shaped glass diaphragm obeys the following equation of motion.

(1) $$m\cdot y^{\prime \prime }\left(t\right)+\beta \cdot y^{\prime }\left(t\right)+k\cdot y\left(t\right)=\left[A+B\cos\left(\theta \right)\right]\cdot S\cdot p_{0}e^{-j\omega t}$$

where *m* and *S* are the mass and area of the diaphragm, *β* and *k* are the damping coefficient and the spring constant for the diaphragm, *P*_0_, ω, and *θ* are the pressure amplitude, the angular frequency, and the incident angle of the plane acoustic wave, y(t), $y^{\prime }\left(t\right)$, and $y^{\prime \prime }\left(t\right)$ are displacement, velocity, and acceleration of the diaphragm center at time *t*. The right item of Equation (1) represents the net force applied to the diaphragm by the incident sound wave, and the factor [A+B cos(θ)] reflects angular dependence of the force difference between the front and rear surfaces of diaphragm. By using *E* and *h* for the Young’s modulus and thickness of the diaphragm and using *W* and *L* for the length and width of each beam of the diaphragm, *k* in Equation (1) can be expressed as the following formula [28]:

(2) $$k=4E\cdot W\cdot h^{3}\cdot L^{-3}$$

*A* and *B* in Equation (1) are dependent on both the diaphragm material used and the depth of the microphone chamber, and they can be treated as two constants for a prepared microphone. In this case, the solution of Equation (1) can be derived as follows:

(3) $$y\left(\omega ,\theta ,t\right)=\frac{\left[A+B\cos\left(\theta \right)\right]\cdot S\cdot p_{0}e^{-j\omega t}}{m\sqrt{{\left(\omega_{0}{}^{2}-\omega^{2}\right)}^{2}+{\left(\omega \beta /m\right)}^{2}}}$$

where $\omega_{0}$ is the resonant angular frequency of the diaphragm and can be written as $\omega_{0}=\sqrt{k/m}$. By substituting $S=\pi r^{2}+4WL$ and m=ρhS into Equation (3), the maximum displacement amplitude ($y_{0}$) of the wheel-shaped diaphragm can be expressed as Equation (4), which is a function of *ω* and *θ*.

(4) $$y_{0}\left(\omega ,\theta \right)=\frac{\left[A+B\cos\left(\theta \right)\right]p_{0}}{\rho h\sqrt{{\left(\omega_{0}{}^{2}-\omega^{2}\right)}^{2}+{\left(\omega \beta /m\right)}^{2}}}$$

where *r* is the radius of the central disc of the diaphragm and ρ is the density of the diaphragm materials. Equation (4) can be used to analyze frequency dependence of the mechanical response of the microphone under the condition of fixing the incident angle of *θ*. Equation (4) can also be used to simulate the directional response of the microphone in the case of fixing the sound frequency of *f* (here *f* = *ω*/2π). It is easily understood from Equation (4) that for a specific sound frequency the displacement amplitude reaches the maximum at θ=0°. With this maximum to normalize the displacement amplitudes at different incident angles, the normalized directional response at the given frequency, *Y*_0_(*θ*), for the microphone can be expressed as Equation (5).

(5) $$Y_{0}\left(\theta \right)=\frac{A+B\cos\left(\theta \right)}{A+B}=\frac{1+\gamma \cos\left(\theta \right)}{1+\gamma }$$

where γ=B/A. Equation (5) can be used to fit the measured directional response data to determine γ for the microphone under test.

The influence of the damping coefficient on the mechanical response of the diaphragm is analyzed based on Equation (4). According to the purchased glass plate and the customized holder for preparing the microphone, the thickness and total diameter of the wheel-shaped diaphragm are fixed to be *h* = 150 μm and *D* = 30 mm, and the rim of the outer ring of the diaphragm is 2.3 mm wide. As a result, the effective diameter of the wheel-shaped diaphragm is 2(*r* + *L*) = 25.4 mm. Only the length (*L*) and width (*W*) of the beam are adjustable for optimal design of the diaphragm. Assuming *A* = *B* = 1 and given *W* = 2.0 mm and *L* = 2.3 mm and *P*_0_ = 1 Pa, the mechanical frequency response of the diaphragm at normal incidence of sound (*θ* = 0°) was calculated with different damping coefficients using Equation (4). Figure 1b shows the calculated results. As the damping coefficient increased, the resonance frequency remained at 1231 Hz, but the maximum displacement amplitude decreased at this frequency.

**Figure 2:**
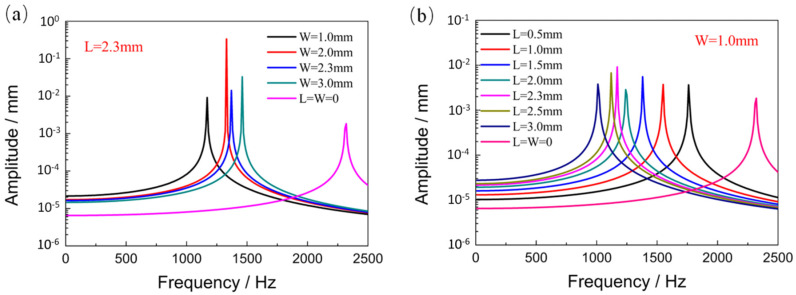
Frequency dependences of the displacement at the center of the wheel-shaped glass diaphragms with different beam widths and lengths obtained with FDTD simulation ((**a**) with a fixed beam length of *L* = 2.3 mm; (**b**) with a fixed beam width of *W* = 1.0 mm).

The impact of four identical beams of the wheel-shaped diaphragm on the frequency response characteristic of the diaphragm was analyzed by using the finite-difference time-domain (FDTD) method. Given the sound pressure to be 1 Pa, the FDTD simulation was performed with different lengths and different widths of the beam. Figure 2a,b show the simulation results, in which the curve obtained with *L* = *W* = 0 represents the mechanical responses at different frequencies for a circular glass diaphragm with a diameter of 1 inch. Compared with this curve, the other curves obtained with *L* > 0 and *W* > 0 showed larger amplitudes in the flat frequency response range below 1000 Hz, which was a contribution of the four glass beams. As the beam width decreased or the beam length increased, the resonant frequency of the diaphragm decreased and the amplitudes of flat frequency response increased. The resonance frequency obtained by FDTD under conditions of *W* = 2.0 mm and *L* = 2.3 mm was 1330 Hz, which was little higher than the resonance frequency of 1231 Hz shown in Figure 1b. The above findings indicate that the wheel-shaped glass diaphragm is suitable for detection of low-frequency sounds and its mechanical sensitivity and flat frequency response range can be optimized by adjusting the geometric parameters of the beam. Considering that the thinner and longer the glass beams, the more likely the wheel-shaped diaphragm is to break during preparation and application, the length and width of each beam of the wheel-shaped glass diaphragm were selected to be *W* = 2.0 and *L* = 2.3 mm. In this case, the displacement amplitude of the diaphragm was approximately 5 times as large as that calculated with *L* = *W* = 0, and the area of holes on the diaphragm occupied 29.3% of the total area of the diaphragm.

### 2.2. Simulation of the Fiber-Optic Microphone

**Figure 3:**
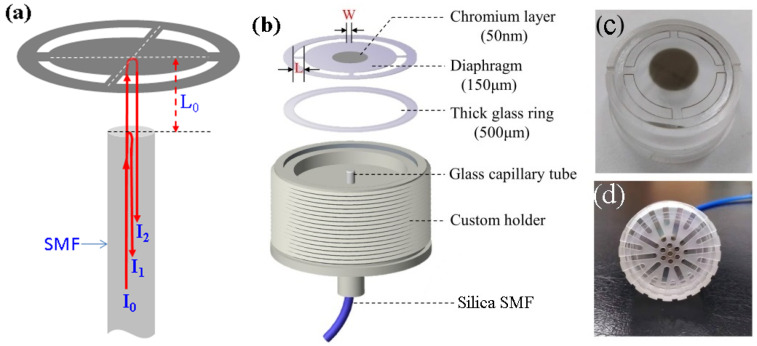
(**a**) Schematic diagram of the Fabry-Perot interferometric principle; (**b**) schematic diagram of the components designed for assembling the fiber-optic microphone head; (**c**) photograph of the wheel-shaped glass diaphragm installed on the holder; (**d**) the prepared microphone head with a protective cap.

Figure 3a schematically shows the Fabry–Perot interferometric principle applied to a fiber-optic microphone. The laser light ($I_{0}$) coupled with the silica single-mode fiber (SMF) was divided into two parts at the fiber endface: the internally reflected light ($I_{1}$) and the transmitted light ($I_{2}$). The latter struck the central reflective area of the wheel-shaped diaphragm and was then reflected back into the SMF, leading to the interference of two light beams ($I_{1}$ and $I_{2}$). The phase difference between the two beams was modulated by $y\left(t\right)=y_{0}\cos\left(\omega t\right)$ and can be expressed as Equation (6):

(6) $$\Delta \phi \left(t\right)=\frac{4\pi n}{\lambda }\left[L_{0}+y\left(t\right)\right]=\frac{4\pi n}{\lambda }\left[L_{0}+y_{0}\cos\left(\omega t\right)\right]$$

where *λ* is the laser wavelength, $L_{0}$ is the distance from the fiber endface to the diaphragm at rest (namely, the initial length of the FP cavity), *n* is refractive index of the medium filling the FP cavity, y(t) is defined above, and $y\left(t\right)=y_{0}\cos\left(\omega t\right)$ ($y_{0}$ is the displacement amplitude at the diaphragm center). Therefore, the output light intensity of the microphone at time *t* can be written as Equation (7):

(7) $$\begin{array}{l} I\left(t\right)=I_{1}+I_{2}+2\sqrt{I_{1}I_{2}}\cos\left\{\frac{4\pi n}{\lambda }\left[L_{0}+y_{0}\cos\left(\omega t\right)\right]\right\}\\ =I_{1}+I_{2}+2\sqrt{I_{1}I_{2}}\left\{\begin{array}{l} \cos\left(\frac{4\pi nL_{0}}{\lambda }\right)\cos\left[\frac{4\pi ny_{0}}{\lambda }\cos\left(\omega t\right)\right]\\ -\sin\left(\frac{4\pi nL_{0}}{\lambda }\right)\sin\left[\frac{4\pi ny_{0}}{\lambda }\cos\left(\omega t\right)\right] \end{array}\right\} \end{array}$$

To make the microphone work at the quadrature point (Q point) of the FP interferometer for high sensitivity and high fidelity, *λ* and $L_{0}$ should satisfy the following relationship:

(8) $$L_{0}=\frac{2m+1}{8n}\lambda ,m=0,1,2,\ldots$$

Once Equation (8) is satisfied, Equation (7) can be simplified as the following formula:

(9) $$I\left(t\right)=I_{1}+I_{2}+2\sqrt{I_{1}I_{2}}\sin\left[\frac{4\pi ny_{0}}{\lambda }\cos\left(\omega t\right)\right]$$

The alternative current (*AC*) term in Equation (9), *I_AC_*(*t*), representing the temporal response of the microphone to sound at angular frequency *ω*, can be expanded into the following power series:

(10) $$I_{AC}\left(t\right)=2\sqrt{I_{1}I_{2}}\left\{\begin{array}{l} \left[\frac{4\pi ny_{0}}{\lambda }\cos\left(\omega t\right)\right]-\frac{1}{3!}{\left[\frac{4\pi ny_{0}}{\lambda }\cos\left(\omega t\right)\right]}^{3}\\ +\frac{1}{5!}{\left[\frac{4\pi ny_{0}}{\lambda }\cos\left(\omega t\right)\right]}^{5}-\ldots \ldots \end{array}\right\}$$

Under the condition that $y_{0}$ does not exceed *λ*/(4π*n*) to guarantee the linear response of the microphone, the high-order power terms in Equation (10) are small and can be ignored. In this case, *I_AC_*(*t*) is approximated as Equation (11):

(11) $$I_{AC}\left(t\right)\approx \frac{8\pi ny_{0}}{\lambda }\sqrt{I_{1}I_{2}}\cos\left(\omega t\right)$$

When the microphone was used to detect airborne sounds, the medium filling the FP cavity was air with *n* = 1 and the endface of silica SMF had a reflectance of *R* = 0.0362. In the case of using the microphone for waterborne sound detection, the medium filling the FP cavity became water with *n* = 1.33 and the reflectance of the fiber endface decreased down to *R* = 0.0025. Given the same *ω* and equal $y_{0}$ for airborne and waterborne sounds, the microphone response to airborne sound was 2.8 times larger than that to waterborne sound according to Equation (11).

Figure 3b schematically shows the components designed for assembling the fiber-optic microphone head. It contains a wheel-shaped glass diaphragm, a glass ring gasket, and a custom holder. The wheel-shaped glass diaphragm was prepared by laser cutting on a 150-μm-thick glass plate, and it consisted of a central glass disc connected to an outer glass ring by four identical glass beams. The glass diaphragm (including the disc and beams beam) had a diameter of 2R=25.4 mm. The glass ring gasket was prepared with a 500-μm-thick borosilicate glass substrate, which was chemically bonded to the outer glass ring of the diaphragm to support the diaphragm from cracking during the microphone head assembly. The specially designed holder with a cylindrical shallow trough and a central through hole was prepared with acrylic block. A glass capillary tube with a silica SMF fixed inside was fixed with epoxy adhesive along the through hole of the custom holder. After chemical bonding of the wheel-shaped glass diaphragm on the thick glass ring, the bonded element was mounted on the top of the custom holder to form an open back air chamber. The fiber tip in the back air chamber points at the center of the glass disc of the diaphragm, forming an extrinsic fiber-optic FPI. The length of the FP cavity defined as the distance between the fiber tip and the disc center is carefully adjusted by monitoring the spectral interference pattern with a spectrometer. To increase the reflectance of the diaphragm, a 50-nm chromium layer was sputtered on the central disc. Note that the chromium layer was chosen as a reflective film based on its excellent adhesion to glass. Figure 3c,d shows the photographs of the microphone head without and with a protection cap.

### 2.3. Experimental Setup

**Figure 4:**
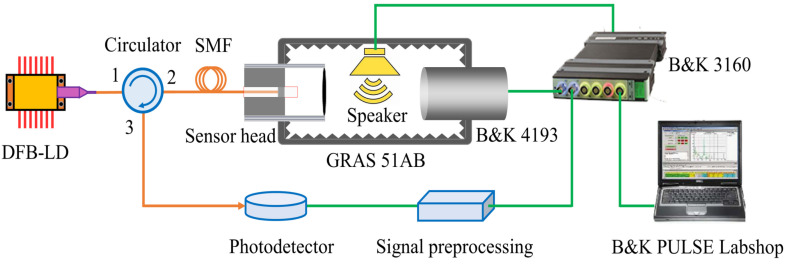
Schematic diagram of the acoustic test system used in this work.

Figure 4 schematically shows the acoustic test system used in this work, which consists of a fiber-coupled DFB laser, a fiber-coupled photodetector, a three-port fiber circulator, a home-made signal preprocessing module, a sound generator (GRAS 51AB), a reference microphone (B&K 4193), a multifunctional module (B&K LAN-3160), and the B&K PULSE Labshop software. The prepared fiber-optic microphone and the DFB laser and the photodetector are connected to the corresponding ports of the fiber circulator to form an extrinsic FP interferometer detection loop. A custom adapter (not shown in Figure 4) is used to connect the fiber-optic microphone head to the sound generator that is controlled with the multifunctional module. The sound pressure from the sound generator was calibrated in real time with the reference microphone. The output signals of the fiber-optic and reference microphones are simultaneously acquired by the multifunctional module. The data analysis is performed by using the PULSE Labshop software.

## 3. Results and Discussions

### 3.1. Determination of the FP Cavity Length of the Fiber-Optic Microphone

The FP cavity length of the fiber-optic microphone was determined by measuring the spectral interference pattern during preparation of the microphone head. To do this, the DFB laser and the photodetector in Figure 4 were replaced by an amplified spontaneous emission (ASE) light source and a high-resolution spectrometer (YOKOGAWA AQ6370B), respectively. Considering that the FP cavity length is easily affected by the ambient temperature, after each adjustment of the FP cavity length, the microphone head was placed in a temperature-controlled thermostat, and then the spectral interference pattern at a given temperature was recorded to determine the cavity length. By selecting the two wavelengths, *λ*_1_ and *λ*_2_, corresponding to the two adjacent peaks (or dips) in the spectral interference pattern, the initial length ($L_{0}$) of the FP cavity at the given temperature is calculated according to Equation (12) [14].

(12) $$L_{0}=\lambda_{1}\lambda_{2}/\left[2\left|\lambda_{1}-\lambda_{2}\right|\right]$$

**Figure 5:**
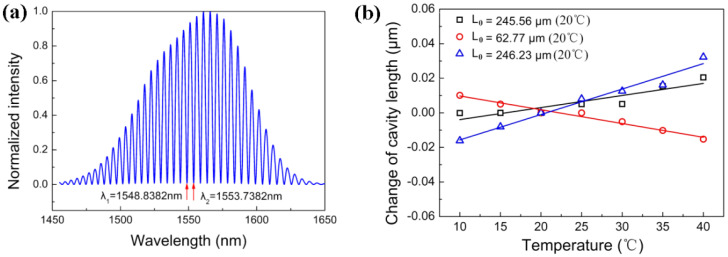
(**a**) Spectral interference pattern of the FPI based fiber-optic microphone measured at 20 °C; (**b**) changes of the FP cavity lengths of the microphone versus ambient temperatures for *L*_0_ = 245.66, 62.77, and 246.23 μm, respectively.

Figure 5a shows the spectral interference pattern of the fiber-optic microphone measured at room temperature (20 °C), which is normalized by the maximum intensity. From this pattern the two wavelengths, *λ*_1_ = 1548.8382 and *λ*_2_ = 1553.7382 nm, corresponding to the two adjacent dips marked with arrows are chosen. By substituting the values of *λ*_1_ and *λ*_2_ in Equation (12), the initial length of the FP cavity is calculated to be $L_{0}=245.56\;$ μm. To investigate the temperature influence on the FP cavity length of the prepared microphone, the spectral interference patterns at different temperatures (10, 15, 25, 30, 35, and 40 °C) were measured, which were then used to determine the cavity lengths at the corresponding temperatures. Figure 5b shows the changes of the FP cavity length measured at different temperatures relative to $L_{0}=245.56\;$ μm obtained at 20 °C (black square). The FP cavity length slightly increases with temperature, and its temperature coefficient is obtained to be 0.7 nm/°C by the linear fitting method. According to our experiments, it is actually difficult to reduce the temperature coefficients to less than 0.7 nm/°C. The further experiments reveal that deviation of the FP cavity length from $L_{0}=245.56\;$ μm enhances temperature effect on it. A positive deviation from $L_{0}=245.56\;$ μm causes the FP cavity to increase with temperature, as shown in blue triangle in Figure 5b, and a negative deviation from $L_{0}=245.56$ μm causes the FP cavity length to decrease with temperature, as shown in red circle in Figure 5b. The above comparison indicates that the prepared fiber-optic microphone with $L_{0}=245.56\;$ μm has a relatively good thermal stability in the range of temperature from 10 to 40 °C. Therefore, the performance of the fiber-optic microphone prepared with $L_{0}=245.56\;$ μm was investigated in this work.

**Figure 6:**
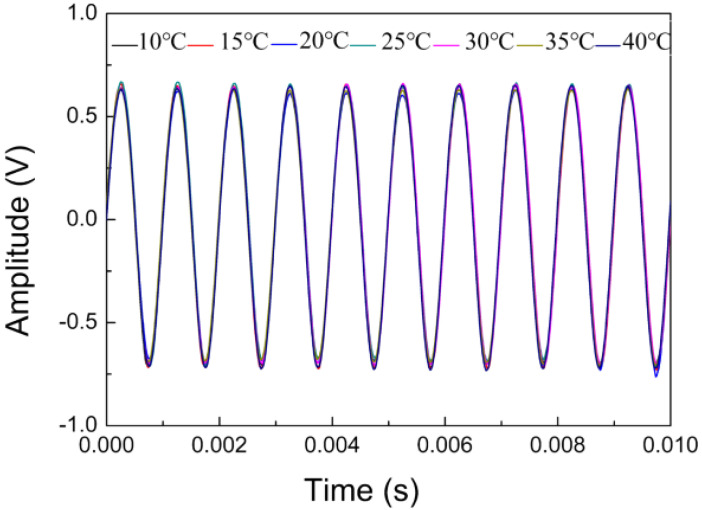
Temporal responses of the fiber-optic microphone to 1 kHz sound measured at different temperatures.

After fixing the initial length of the FP cavity at $\;L_{0}=245.56\;$ μm, the temporal response of the prepared microphone to airborne sound was measured at different ambient temperatures. To do this, a DFB laser of 1550 nm wavelength was used as the light source of the microphone, and the sound generator is placed in the thermostat along with the B&K 4193 microphone and the fiber-optic microphone. The frequency and pressure of the incident sound from the sound generator is 1 kHz and 0.33 Pa. Figure 6 displays the temporal response curves measured at seven temperatures (10, 15, 20, 25, 30, 35, and 40 °C). All these curves are almost identical to each other, indicating that the FP cavity length of the prepared microphone indeed presents good thermal stability. In addition, it is worth noting that the wheel-shaped glass diaphragm can quickly equalize the air pressure inside and outside the back chamber of the prepared microphone when it experiences a rapid thermal shock or a sudden change in air pressure.

### 3.2. Responses of the Microphone to Airborne Sounds

**Figure 7:**
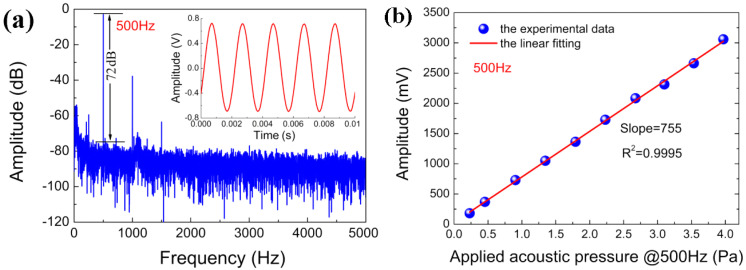
(**a**) the microphone response in frquency domain to an airborne sound with a freqneucy of 5oo Hz and a pressure of 1 Pa (inset: time domian response curve measured with the microphone); (**b**) the response amplitude versus applied acoustic pressure at 500 Hz.

Using the acoustic test platform shown in Figure 4, the temporal response of the prepared fiber-optic microphone to a monotone sound was measured at normal incidence. The incident sound has a frequency of 500 Hz and a pressure of 1 Pa. The measured temporal response is shown in the inset of Figure 7a, and the corresponding FFT result with a frequency resolution of Δ*f* = 1 Hz is displayed in Figure 7a, in which there are the fundamental peak at 500 Hz and the second harmonic peak at 1000 Hz and the third harmonic peak at 1500 Hz. The presence of harmonic signals implies that the fiber-optic microphone under test does not work at the quadrature point and Equation (8) is not satisfied [29]. The signal-to-noise ratio (SNR) for the fundamental signal is 72 dB relative to 1 V. Therefore, the minimum detectable pressure (MDP) for the microphone under test is calculated to be 251 μPa/Hz^1/2^ based on the following equation [30].

(13) $$\mathrm{MDP}=P{\left(10^{\frac{SNR}{20}}\sqrt{\Delta f}\right)}^{-1}$$

From the frequency response curve measured below, it is seen that the above MDP value corresponds to the minimum detectable pressure of the microphone in the flat frequency response range, and the MDP will be greatly reduced in the resonance frequency range.

The pressure sensitivity at 500 Hz for the microphone under test was investigated by measuring its response amplitudes at different sound pressures. As shown in Figure 7b, the amplitude linearly increases with sound pressure, and the slope representing the pressure sensitivity of the microphone is equal to 755 mV/Pa. This high sensitivity is attributed to the fiber-optic FP interferometry that always detects the maximum displacement of the diaphragm.

**Figure 8:**
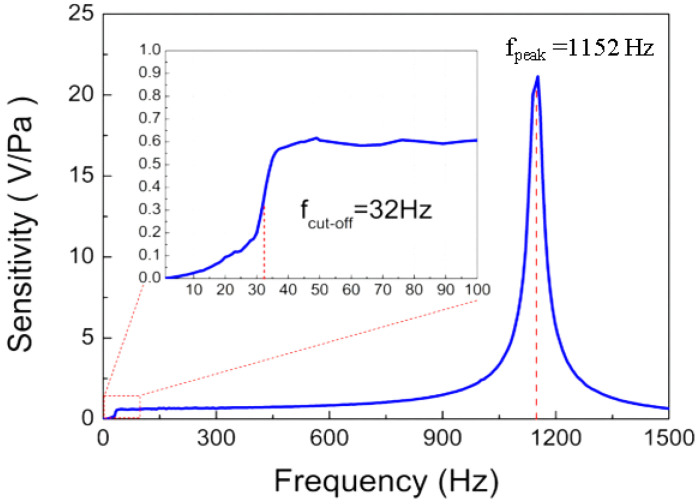
Frequency responses of the fiber-optic microphone (inset: an enlarged view of the low-frequency regime).

The frequency response of the prepared fiber-optic microphone was investigated under the condition of the normal incidence. Figure 8 shows the measured frequency response curve, which includes a steep slope in the low frequency regime, a plateau located between 50 and 800 Hz and a narrow resonance band with the peak frequency of $f_{0}=1152$ Hz. The plateau indicates that the microphone presents a relatively flat frequency response in the frequency range of 50 to 800 Hz. The inset is a magnified view of the steep slope, from which the 3 dB cut-off frequency is determined to be 32 Hz. The measured resonance frequency of $f_{0}=1152$ Hz is slightly smaller than the numerically calculated value of $f_{0}=1231$ Hz, attributed to the reflective chromium layer deposited on the central disc of the diaphragm, which is not considered in the calculation process. The narrow resonance band shown in Figure 8 presents a full width at half maximum (FWHM) of Δf=55  nm, and the quality factor (Q) of the prepared microphone is calculated as Q = $f_{0}$/Δf = 21. This resonance characteristic of the microphone is remarkable, attributed to the open chamber structure that effectively reduces the air damping. The damping coefficient is calculated to be *β* = 0.024 according to the formula *β* = 1/(2Q), which is much smaller than that for conventional electret condenser microphones (typically Q = 1 for pressure-field electret microphones). The sharp resonance peak in Figure 8 suggests that the fiber-optic microphone prepared with a wheel-shaped glass diaphragm is suitable for sensitive detection of monotonic sound with a frequency of interest (for example, detection of photoacoustics). This is so because the wheel-shaped glass diaphragm can be precisely designed through simulation guidance to make its resonance peak at the wanted frequency.

**Figure 9:**
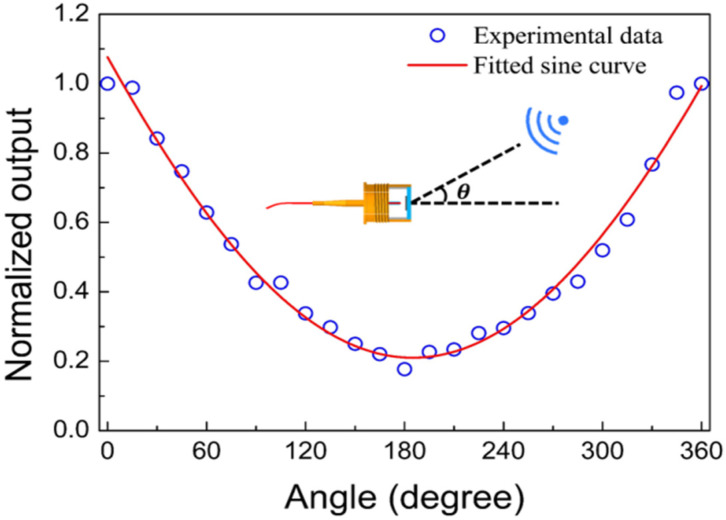
Directional response curve measured for the microphone under test (for comparison, the curve is normalized with the maximum output intensity measured at *θ* = 0°).

The directional response of the microphone was investigated in an anechoic room containing a loudspeaker and a program-controlled horizontal rotating stage. The microphone under test was mounted on the rotating stage, making the normal of the wheel-shaped glass diaphragm parallel to the plane of the stage. The loudspeaker is located 1 m away from the rotating stage, and they are at the same height. By facing the diaphragm of the microphone toward the loudspeaker, the initial incident angle of the sound wave from the loudspeaker is defined as *θ* = 0. Measurement was performed by fixing the frequency of the incident sound at *f* = 1 kHz and then step-by-step rotating the stage in an interval of 15 degree. The sound pressure at the fiber-optic microphone location was calibrated to be *P* = 1 Pa using the B&K 4193 electret microphone. The response amplitude of the fiber-optic microphone was recorded at each rotating step. Figure 9 shows the experimental results, in which the response amplitudes were normalized with its maximum. Obviously, the microphone response depends on the incident angle of the sound wave, and its minimum and maximum response amplitudes appear at *θ* = 180° and *θ* = 0°, respectively. It is worth noting that the directional response curve in Figure 9 corresponds to a cardioid microphone. Fitting the experimental data in Figure 9 with Equation (5) results in *γ* = 0.75.

### 3.3. Preliminary Investigation of the Microphone Response to Waterborne Sounds

**Figure 10:**
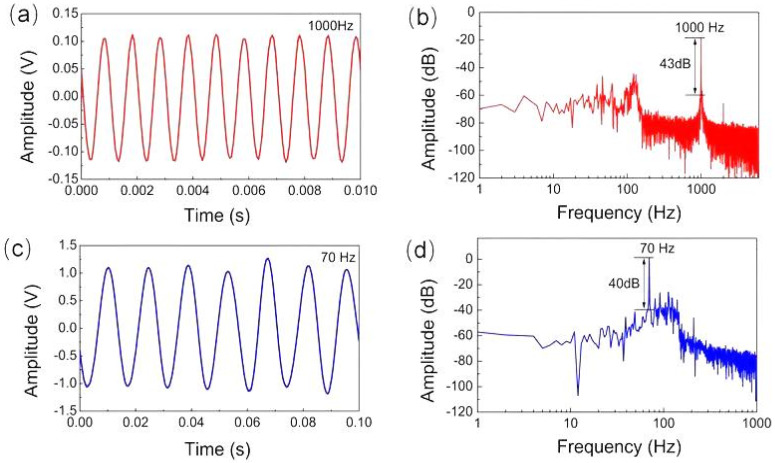
Temporal responses of the fiber-optic microphone to waterborne sounds and the corresponding FFT spectra, (**a**,**b**) with 1k Hz waterborne sound, (**c**,**d**) with 70 Hz waterborne sound.

**Figure 11:**
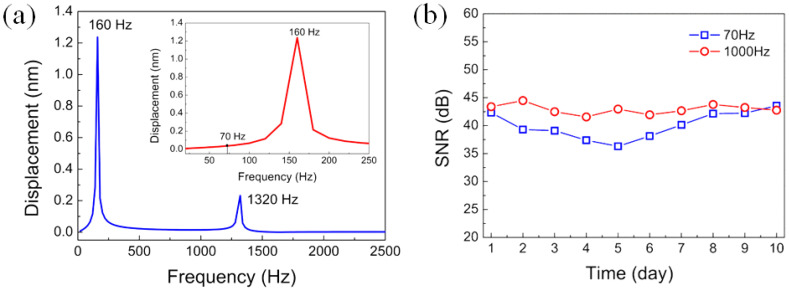
(**a**) Frequency response to waterborne sound of the wheel-shaped glass diaphragm-based fiber-optic microphone simulated at 1 Pa pressure, (**b**) SNRs of the fiber-optic microphone at 70 and 1000 Hz measured underwater on different days.

The integration of the fiber-optic transducer and the open back chamber into the microphone enabled it to be used under water as a hydrophone. To demonstrate this unique capability, a simple experimental setup was constructed using a water tank filled with tap water. A closed box with a loudspeaker inside was kept under water in the tank for generating waterborne sound of single frequency, and the microphone head was directly immersed in water without any protection. The microphone response was then measured at a higher frequency of 1000 Hz and a lower frequency of 70 Hz. Note that 70 Hz sound is the lowest frequency sound without distortion the loudspeaker used can generate. Figure 10a shows the measured temporal response of the microphone to 1000 Hz waterborne sound, which was processed by fast Fourier transform (FFT) algorithm to give the frequency-domain signal in dB (relative to 1 V). As shown in Figure 10b, the amplitude peak appeared at exactly 1000 Hz frequency with the peak value of −19.1 dB relative to 1 V, and the SNR was approximately 43 dB. Figure 10c displays the temporal response of the microphone measured at 70 Hz, and the corresponding FFT spectrum is shown in Figure 10d. The signal in frequency domain located at 70 Hz with the peak value of 0.97 dB and the SNR was 40 dB. Although the signal amplitude at 70 Hz was larger than that at 1000 Hz, the SNR at 70 Hz was smaller than that at 1000 Hz. The comparison indicates a better performance of the microphone at 1000 Hz for underwater detection. Because a standard hydrophone was not available in the present work, the waterborne sound pressures at different frequencies could not be calibrated, making the frequency response characteristic of the prepared fiber-optic microphone unable to be measured under water. Instead, simulation of the frequency response of the microphone to waterborne sounds of 1 Pa pressure was carried out using the same geometric parameters of the wheel-shaped glass diaphragm as shown in Section 2.1. Figure 11a shows the simulation curve, which includes two resonant peaks at 160 and 1330 Hz, respectively. The resonant peak at 1330 Hz was a common feature of the frequency response of the microphone in air and underwater, but the fundamental resonance peak at 160 Hz is a characteristic of the microphone’s response only to waterborne sounds. The calculated displacement amplitude at 70 Hz was 0.041 nm, larger than 0.015 nm obtained at 1000 Hz. This simulation result was very helpful for us to understand why the measured response of the microphone at 70 Hz was much larger than that at 1000 Hz.

To investigate the long-time stability of the microphone under water, its temporal responses to 70 and 1000 Hz waterborne sounds were measured daily over ten days. The time-domain results obtained at different days have no obvious difference, which are similar to those shown in Figure 10a,c. Only the SNR values at each frequency exhibited small fluctuation between 37 and 45 dB, as shown in Figure 11b. These preliminary experimental results revealed that the fiber-optic microphone prepared with a wheel-shaped glass diaphragm can stably work under water for a long time without obvious performance degradation. This opens a new approach to underwater detection using the fiber-optic microphone as a hydrophone. When a standard hydrophone is available, an in-depth investigation of the underwater performance of the prepared fiber-optic microphone will be carried out. The performance characteristics of existing and our fiber-optic microphones are summarized in Table 1, from which it can be seen that our microphone outperformed those microphones in terms of sensitivity and low-frequency waterborne sound detection.

## 4. Conclusions

A fiber-optic microphone with the wheel-shaped glass diaphragm was theoretically and experimentally demonstrated in this work. The wheel-shaped glass diaphragm was designed with simulation and prepared by laser cutting on a 150-μm-thick glass substrate, and the microphone was fabricated based on fiber-optic FP interferometry, with an optimized length of the FP cavity. The combination of the fiber-optic FPI transducer and the glass diaphragm made the microphone electromagnetically and chemically robust, offering the microphone good applicability in harsh environments. The wheel-shaped glass diaphragm opened the back chamber of the microphone, resulting in three unique features: (1) it eliminated the background pressure drifting problem, consequently improving the thermal stability of the microphone; (2) it suppressed air damping on the diaphragm, thereby increasing sensitivity of the microphone; (3) it allowed the microphone to be easily and safely used for underwater sound detection. The microphone operates at 1550 nm wavelength with the intensity-interrogating manner, showing good performance in air and under water. According to experimental results, the prepared fiber-optic microphone in air was relatively stable in a range of temperature from 10 to 40 °C, having a lower frequency detection limit of 32 Hz and a fundamental resonance peak at 1152 Hz with a quality factor of Q = 21. Such a large quality factor makes the microphone quite suitable for photoacoustic detection. The sensitivity of the microphone to airborne sound of 500 Hz frequency was 755 mV/Pa and the corresponding MDP was 251 μPa/Hz^1/2^. In response to underwater sounds, the fundamental resonance peak of the microphone moved to a lower frequency of 160 Hz. A large response of the microphone to 70 Hz waterborne sound was experimentally obtained, demonstrating its applicability as a hydrophone. To the best of our knowledge, this is the first report on a wheel-shaped glass diaphragm-based fiber-optic microphone with high performance and multifunctional applications.

## Figures and Tables

**Table 1:** Performance characteristics of existing and our fiber-optic microphones.

Diaphragm Type	Diaphragm Thickness	Sensitivity and MDP	Frequency Response Range	Underwater Detection Capability
Corrugated silver [14]	~5 μm	52 nm/Pa, 86.97 μPa/Hz^1/2^ @1 kHz	200 Hz~1 kHz	No
PDMS [21]	9.6 μm	427 mV/Pa	10 Hz~50 Hz	No
Graphene [31]	100 nm	1100 nm/kPa, 60 µPa/Hz^1/2^ @ 10 kHz	0.2 Hz~22 kHz	No
Silicon [18]	3 μm	94 mV/Pa @ 1 kHz	100 Hz~2.5 kHz	No
Stainless steel cantilever [32]	5 μm	211.2 nm/Pa, 5 μPa/Hz^1/2^ @ 1 kHz	below 2 kHz	No
Wheel-shaped glass (this work)	150 μm	755 mV/Pa, 126 μPa/Hz^1/2^ @ 500 Hz	32 Hz~800 Hz	Yes
